# Comparative Evaluation of Microleakage of Flowable Composite Resin Using Etch and Rinse, Self-Etch Adhesive Systems, and Self-Adhesive Flowable Composite Resin in Class V Cavities: Confocal Laser Microscopic Study

**DOI:** 10.3390/ma15144963

**Published:** 2022-07-16

**Authors:** Ekta Varma Sengar, Sanjyot Mulay, Lotika Beri, Archana Gupta, Thamer Almohareb, Sultan Binalrimal, Ali Robaian, Maha A. Bahammam, Hammam Ahmed Bahammam, Sarah Ahmed Bahammam, Bassam Zidane, Nassreen H. Albar, Shilpa Bhandi, Deepti Shrivastava, Kumar Chandan Srivastava, Shankargouda Patil

**Affiliations:** 1 Department of Conservative Dentistry and Endodontics, Dr. D. Y. Patil Dental College & Hospital, Dr. D. Y. Patil Vidyapeeth, Pimpri, Pune 411018, India; ektavarma1993@gmail.com (E.V.S.); sanjyot.mulay@dpu.edu.in (S.M.); lotika.lb@gmail.com (L.B.); 2 Independent Researcher, Pune 411018, India; archanaanshumangupta@gmail.com; 3 Restorative Dental Science Department, College of Dentistry, King Saud University, Riyadh 11451, Saudi Arabia; talmohareb@ksu.edu.sa; 4 Restorative Department, College of Dentistry, Riyadh Elm University, Riyadh 13244, Saudi Arabia; sultan@riyadh.edu.sa; 5 Conservative Dental Sciences Department, College of Dentistry, Prince Sattam Bin Abdulaziz University, Alkharj 16245, Saudi Arabia; ali.alqahtani@psau.edu.sa; 6 Department of Periodontology, Faculty of Dentistry, King Abdulaziz University, Jeddah 21589, Saudi Arabia; mbahammam@kau.edu.sa; 7 Executive Presidency of Academic Affairs, Saudi Commission for Health Specialties, Riyadh 11614, Saudi Arabia; 8 Department of Pediatric Dentistry, College of Dentistry, King Abdulaziz University, Jeddah 21589, Saudi Arabia; habahammam@kau.edu.sa; 9 Department of Pediatric Dentistry and Orthodontics, College of Dentistry, Taibah University, Medina 42353, Saudi Arabia; sbahammam@taibahu.edu.sa; 10 Restorative Dentistry Department, Faculty of Dentistry, King Abdulaziz University, Jeddah 21589, Saudi Arabia; bzidane@kau.edu.sa; 11 Department of Restorative Dental Sciences, Division of Operative Dentistry, College of Dentistry, Jazan University, Jazan 45412, Saudi Arabia; nalbar01@gmail.com (N.H.A.); shilpa.bhandi@gmail.com (S.B.); 12 Department of Cariology, Saveetha Dental College & Hospitals, Saveetha Institute of Medical and Technical Sciences, Saveetha University, Chennai 600077, India; 13 Department of Preventive Dentistry, College of Dentistry, Jouf University, Sakaka 72345, Saudi Arabia; sdeepti20@gmail.com; 14 Department of Oral & Maxillofacial Surgery & Diagnostic Sciences, College of Dentistry, Jouf University, Sakaka 72345, Saudi Arabia; 15 Department of Maxillofacial Surgery and Diagnostic Sciences, Division of Oral Pathology, College of Dentistry, Jazan University, Jazan 45412, Saudi Arabia; 16 Centre of Molecular Medicine and Diagnostics (COMManD), Saveetha Dental College & Hospitals, Saveetha Institute of Medical and Technical Sciences, Saveetha University, Chennai 600077, India

**Keywords:** restorative material, polymer, composite resin, flowable composite, dental leakage, dentin bonding agent

## Abstract

The essential factor in determining the preservation of restoration is the marginal seal. Restoring cervical lesions with a resin composite has always been a challenge. Composite resins with various viscosities and different bonding systems are being researched to reduce the microleakage. Confocal laser scanning microscopy (CLSM) is the latest non-destructive technique for visualizing the microleakage. **Objectives:** To evaluate and compare the microleakage of Universal Flo composite resin (G-aenial) using etch and rinse adhesive system ER-2 steps (Adper Single Bond 2), self-etch adhesive system SE-1 step (G-Bond), and self-adhesive flowable composite resin (Constic) in Class V cavities using a confocal laser scanning microscope. **Materials and Method**: Class V cavities were prepared on 27 caries-free human extracted premolar teeth on the buccal and lingual surfaces with standardized dimensions of 2 mm height, width 4 mm, and a depth of 2 mm. After the cavity preparation, all teeth were randomly divided into three groups, namely Group-I: G-aenial Universal Flo with Single Bond 2 (*n* = 9 teeth); Group-II: G- aenial Universal Flo with G-Bond (*n* = 9 teeth), and Group-III: Constic (*n* = 9 teeth). The prepared and restored specimens were then subjected to thermocycling for 500 cycles in a water bath at 5 °C and 55 °C with a dwelling time of 30 s. The specimens were placed in 0.6% aqueous rhodamine dye for 48 h. Sectioning was carried out bucco-lingually and specimens were evaluated for microleakage under a confocal laser scanning microscope. **Results:** There was a significant difference (*p* = 0.009) in microleakage when comparing total etch and rinse, specifically between Adper Single Bond 2 ER-2 steps (fifth generation) and self-adhesive flowable composite resin, which is Constic. There was more microleakage in the self-etch bonding agent, particularly G-Bond, SE-1 step (seventh generation), when compared to ER-2 steps (fifth generation bonding agent); however, the results were not statistically significant (*p* = 0.468). The self-adhesive flowable composite resin showed more microleakage than SE-1 step and ER-2 steps. **Conclusions:** None of the adhesive systems tested were free from microleakage. However, less microleakage was observed in the total etch and rinse, especially Adper Single Bond 2 (ER-2 steps), than the self-etch adhesive system SE-1 step and self-adhesive flowable composite resin. **Clinical significance**: Constant research and technological advancements are taking place in dentin adhesives to improve the marginal seal. This has led to the evolution of total acid-etching dentin bonding agents termed as etch and rinse (ER)-2 steps (fifth generation dentin bonding agents) and self-etching (SE) 2 steps, and SE-1 step dentin bonding agents termed as the sixth and seventh generation bonding agents, respectively.

## 1. Introduction

Restorative dentistry has always thrived to obtain biocompatible restorations with superior aesthetic demand and a good marginal seal [1]. In today’s clinical practice, composite resins have gained wide acceptance due to their high aesthetic quality [2].

Late 1996 led to the development of flowable composites. Their lower filler content, low viscosity and coefficient of thermal expansion being similar to that of the tooth structure made them widely used for restoring class V cavities [3]. Composite resins have progressed from macro-filled to micro-filled restoratives and from hybrids to micro-hybrids, and now the introduction of new nano-filled flowable composite and self-adhesive flowable resins has opened doors in dentistry. These are composed of nanocluster filler particle and nanomer, and thus have a better physical property and less marginal leakage [4].

Despite continuous development in the field of resin material, polymerization shrinkage still occurs. This is the crucial factor for determining the marginal integrity of the restoration [5]. The incidence of cervical lesions are on the rise [5,6]. Present literature states that the reason for the development of these lesions cannot be stated under one etiological factor, but is multifactorial in origin [7,8].

Abfraction is considered as a pathological loss of tooth structure in the cervical region of the tooth when eccentric and excessive occlusal forces act on the teeth [9,10]. Restoring cervical lesions with composite resins is always challenging, chiefly due to gingival margin lying in cementum, leaving no enamel margin for bonding. Isolation is difficult at gingival margins, which may lead to microleakage, and the outcome will be sensitivity, bacterial invasion and failure in the restoration. The longevity and success of such restorations can be assured by establishing a complete seal [11,12,13]. An adequate seal is a fundamental goal to obtain in either direct or in indirect adhesive restorations [14].

Despite continuous development in the field of resin material, polymerization shrinkage still occurs, which is the crucial factor for determining the marginal integrity of the restoration [15]. An increase in aesthetic demands and ease of operative procedures has also led to research and development in newer bonding systems [16]. Approximately 20 years ago, in 1960, Castan began an evolution regarding the concept of the self-etching approach that has reached a hallmark [17]. Recently, the new dentin bonding agents permit the use of acid, primer and bonding agents all together concomitantly [18]. Dentin demineralization followed by monomer penetration into the porosities are the mechanism of action [19]. Thus, these systems are considered to be simple and time- saving [20].

Although total etch adhesives are considered as the ‘gold standard’ for many years [21], studies have shown that total adhesives are incapable of preventing nanoleakage, and post-operative sensitivity has been reported [22]. The increase in the number of clinical steps is technique-sensitive and also time-consuming [23]. Therefore, the current trend is to transpose towards self-etch adhesives.

Currently, self-adhesive flowable composites are developed in order to overcome the time consumption by traditional materials. Bringing novel perception to restorative dentistry [24], they combine the merits of adhesive and restorative resin in a single technology, making new changes in the field of restorative dentistry and claiming to have less microleakage and a high bond strength [25].

Self-etch flowable composite resin requires no etching and bonding as a separate step. It claims that the material provides a good sealing ability, reduces the chair side time and is also less technique-sensitive [26].

Confocal laser scanning microscopy (CLSM) has been used for the three-dimensional evaluation of an object and obtaining a clear image. CLSM has powerful software that displays and analyzes 3D data, and images obtained have a greater sensitivity contrast and high resolution. In addition, optical sectioning giving three-dimensional reconstruction avoids the physical sectioning of specimen [27].

Hence, there is a need to study and evaluate the bonding efficacy of these newly developed self-etch adhesives and self-etch composites in comparison to etch and rinse adhesives in Class V cavities. The commercially available branded materials of a reputed company were selected for this purpose. Progress in the field of adhesive dentistry has given us a unique, less researched, all-in-one composite material called Constic.

This study aims to evaluate and compare the microleakage of Universal Flo composite resin (G-aenial) using an etch and rinse adhesive system, i.e., fifth generation ER-2steps (Adper Single Bond 2), self-etch adhesive system, i.e., seventh generation SE-1 step (G-Bond) and self-adhesive flowable composite resin (Constic) in Class V cavities using a confocal laser scanning microscope.

The null hypothesis states that there is no difference in the microleakage amongst the etch and rinse adhesive system ER-2steps (Adper Single Bond 2), self-etch adhesive system SE-1 step (G-Bond) and self-adhesive flowable composite resin (Constic) in Class V cavities.

## 2. Materials and Methods

This experimental in vitro research study was carried out in Dr. D.Y.Patil Dental College and Hospital, Pimpri, Pune. Prior approval from ethics committee was taken (DYPDCH/696/2016/20).

Twenty-seven human-extracted premolar teeth were selected for the study, and were extracted for orthodontic reasons. Maxillary and mandibular teeth were selected. After removal of residual tags, the specimens were cleaned with pumice, kept in normal saline and used in less than 2 months. Class V cavities were prepared on buccal and lingual surfaces of the teeth.

Cavities were prepared with uniform dimensions of height 2 mm, width 4 mm and depth of 2 mm using No. 329 fissure bur (Mani INC, Utsunomiya, Japan), and airotor handpiece (NSK, Shinagawa City, Japan), and diagnostic instruments such as single-end straight probe (GDC, Bangalore, India), William periodontal probe (GDC, Bangalore, India) and tweezer (GDC, Bangalore, India) were used.

After cavity preparation, all teeth were randomly divided into three groups with different adhesive systems to evaluate microleakage of each under flowable composite resin.

- Group I—G-aenial Universal Flo with Adper Single Bond2 (*n* = 9 teeth)
  (Etch and rinse adhesive system, ER-2 steps);
- Group-II—G-aenial Universal Flo with G-Bond (*n* = 9 teeth)
  (Self-etch adhesive system, SE-1 step);
- Group-III—Constic
  (Self-adhesive flowable composite resin) (*n* = 9 teeth).

In Group I, after preparation of Class V cavities, they were etched for 15 s using 37% phosphoric acid etching gel (3M ESPE, USA) and washed off for 10–15 s, and the cavities were dried until moist cavity surface was visible. Then, one coat of Adper Single Bond 2 (ER-2 steps-fifth generation bonding agent, 3M ESPE, Schiller Park, IL, USA) was applied on the inner surface of the cavity using an applicator brush (Denbur, Westmont, IL, USA) and air dried, followed by another coat, which was light cured using LED light curing unit (Confident, Bengaluru, India) for 10 s. The cavity was then restored with G-aenial universal Flo (GC Corp., Tokyo, Japan), and then again light cured for 20 s.

In Group II, after preparation of Class V cavities, two coats of G-Bond (SE-1 step-seventh generation bonding agent, GC Corp., Tokyo, Japan) was applied on the inner surface of the cavity using an applicator brush, air dried and cured for 10 s. The cavity was then restored with G-aenial universal Flo (GC Corp., Tokyo, Japan) and then cured for 20 s.

In Group III, after preparation of Class V cavities, it was restored with Constic (DMG, Davis, CA, USA). The resin composite surface was rubbed with the help of a brush and then cured for 20 s each.

The restored cavities were polished using composite polishing kit (Shofu, San Marcos, CA, USA). The prepared and restored specimens were then subjected to thermocycling for 500 cycles in water bath (Thermocycler 1100 SD, Mechatronik, Pleidelsheim, Germany) at 5°and 55 °C with a dwell time of 30 s. Nail varnish was applied, except on restorative material and 1 mm area around it. The specimens were placed in 0.6% aqueous rhodamine dye (HiMedia, Mumbai, India) for 48 h. The specimens were then rinsed with water and sectioned bucco-lingually through the center of restoration using slow-speed diamond disc (Shofu, San Marcos, CA, USA) on micromotor (Marathon-4, Seoul, Korea). A total of 36 sections were thus obtained in each group. Each cavity section was evaluated for microleakage by two observers using confocal laser scanning microscope (Zeiss LSM 710, Oberkochen, Germany) at 10× magnification.

The scores were given to the images according to the scoring criteria of Silveira de Araujo et al. [1].

- Score 0—No dye penetration.
- Score 1—Penetration involving half the occlusal and/or gingival wall.
- Score 2—Penetration involving more than half the occlusal and/or gingival wall.
- Score 3—Penetration involving up to the axial wall.

Statistical analysis was carried out using Kruskal–Wallis and Mann–Whitney U test. The *p* value was set at (0.025). The results were processed and analyzed using SPSS software version 19—SPSS Inc., Chicago, IL, USA.

## 3. Results

**Figure 1:**
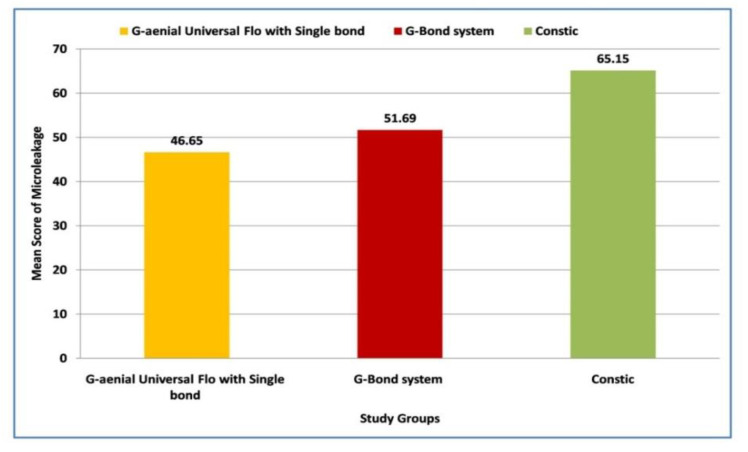
Comparison of mean values of microleakage in class V cavities with Single Bond and G-Bond system and Constic, using G-aenial Universal Flo.

In statistical analysis, mean value/ score for microleakage was 46.65, 51.69 and 65.15 in Group I, II and III, respectively, with the highest microleakage observed in Group III compared to other groups (Figure 1).

**Figure 2:**
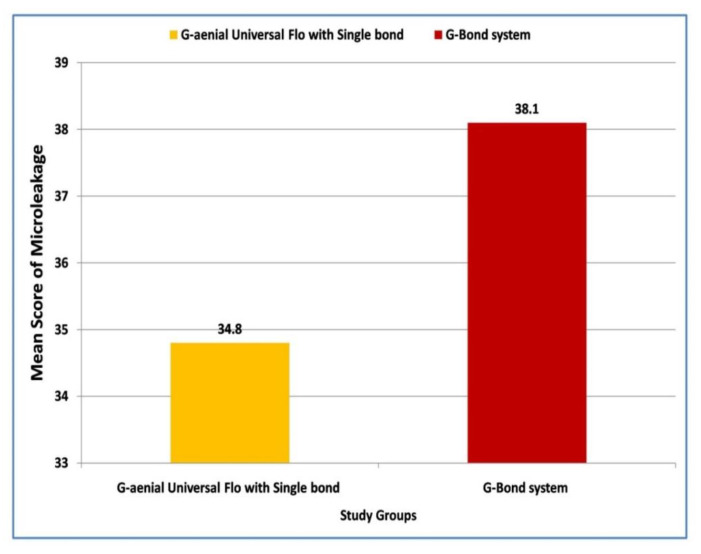
Comparison of mean values of microleakage in class V cavities with Single Bond and G-Bond system, using G-aenial Universal Flo.

**Figure 3:**
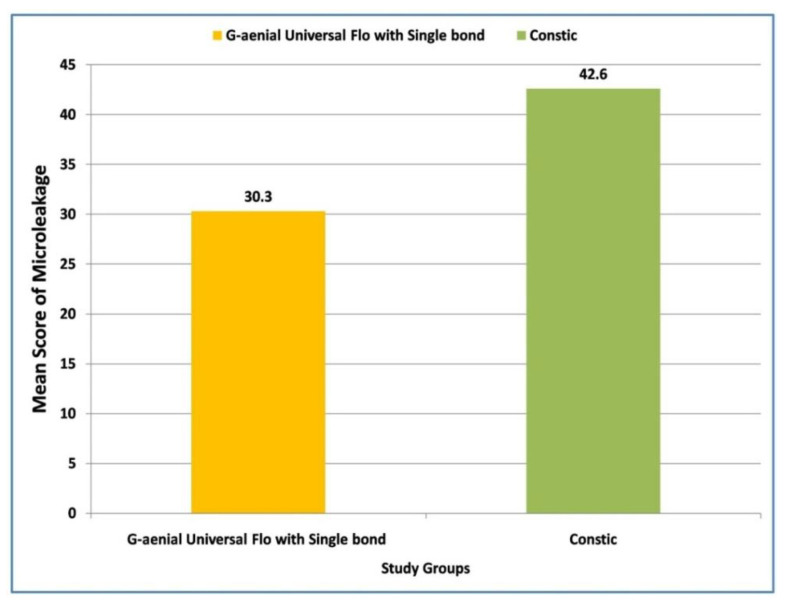
Comparison of mean values of microleakage in class V cavities with Single Bond and Constic, using G-aenial Universal Flo.

**Figure 4:**
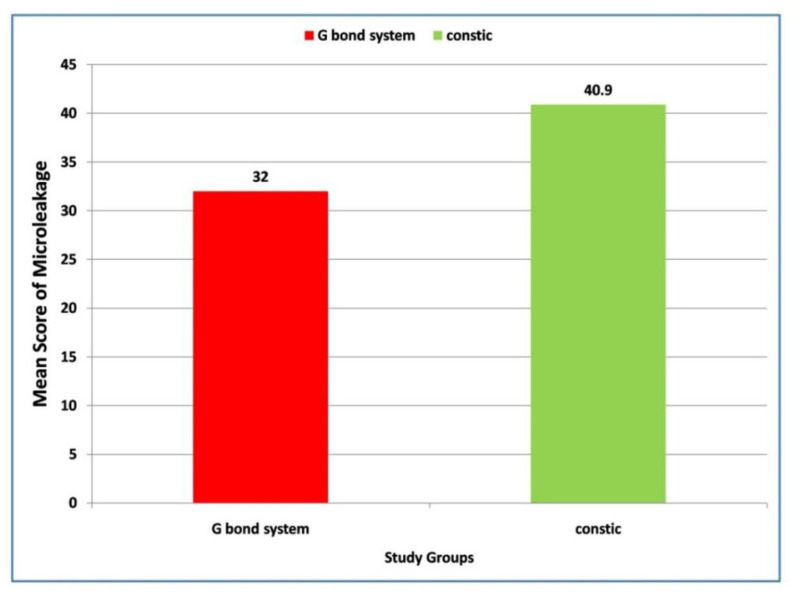
Comparison of mean values of microleakage in class V cavities with G-Bond system and Constic using G-aenial Universal Flo.

Later pair-wise comparisons were carried out using the Mann–Whitney U test. Regarding the intergroup comparison, a higher (38.18 vs. 34.82) although non-significant (*p* = 0.468) microleakage score was found in Group II when compared with Group I (Figure 2). However, a significantly (*p* = 0.009) higher microleakage (42.67 vs. 30.33) was reported in Group III in contrast to Group I (Figure 3). Lastly, a non-significant (*p* = 0.058) difference in microleakage was observed between Group II and Group III (Figure 4).

## 4. Discussion

Restorative dentistry endeavors to meet the aesthetic demands. Today, composite resins play a pivotal role in order to meet these demands. Other than aesthetics, composite resins have many more advantages, such as the conservation of the tooth structure, a low thermal conductivity and being repairable [2]. The longevity of composite resin restoration is, however, related to its marginal integrity. Marginal integrity is influenced by numerous factors, such as tooth preparation, type of restorative material used, restorative technique and also the finishing procedure [28,29].

However, as adhesion to enamel is a predictable entity, it is difficult for it to achieve an adequate bond to dentin. Various technological advancements of dentin adhesives have taken place over time to overcome this challenge. This has led to the evolution of fifth generation total acid-etching dentin bonding agents (ER-2 steps) and self-etching dentin bonding agents termed as the sixth (SE-2 steps) and seventh generation (SE-1step) bonding agents [30].

Adper Single Bond is ER-2 steps (fifth generation total acid etching dentin bonding agent). It consists of 2-hydroxyethyl methacrylate (HEMA), diurethanedimethacrylate, ethyl alcohol, glycerol 1,3-dimethacrylate Bis-GMA, silane-treated silica, itaconic acids and copolymers of acrylic acids [31]. Its composition involves the combination of the functions of a primer and components of an adhesive of a three-step conventional adhesive system, and its solvent consists of alcohol and water. The diffusion of the adhesive into the dentinal tubules is improved by the alcohol present in the solvent, which helps in enhancing the adhesion. The mechanism of action taking place is that the moisture present in the dentinal tubules draws the alcohol into and within the dentinal tubules along with the resin. From the substrate, the moisture content and alcohol vaporize, leaving the resin behind [32].

G-Bond is a SE-1 step (seventh generation) all-in-one self-etching adhesive system. It consists of 4-methacryloxethyl trimellitate anhydride, urethane dimethacrylate (UDMA), triethyleneglycoldimetacrylate, acetone and distilled water [11]. Vinay et al. studied the microleakage of G-Bond in class V cavities. The study showed that, when the samples were treated with this bonding agent, there was no exposure of the collagen fibers, and a slight decalcification was seen at dentin [29]. The functional monomers in the G-Bond react at the nano level with the hydroxyapatite crystals and form insoluble calcium. It has been shown that the G-bond is expected to be more durable and stronger. Five percent of the G-Bond consists of fillers, which seals the dentinal tubules and also decreases pulpal sensitivity [33]. A close marginal adaptation and less gap formation between the adhesive and dentin causes less microleakage and prevents microbial invasion, further leading to an increase in the longevity of restoration.

Constic, a self-adhesive flowable composite resin, was used for this study. Constic consists of 10-methacryloyloxydecyl dihydrogen phosphate. MDP, in a comparison with glycerol phosphate dimethacrylate (GPD), which a monomer used in other selfetch adhesive resins, is found to hold on to a greater number of hydrophobic spacer chains [34]. MDP has shown to form strong bonding with the hydroxyapatite crystals, forming stable 10-MDP-Ca salts.

**Figure 5:**
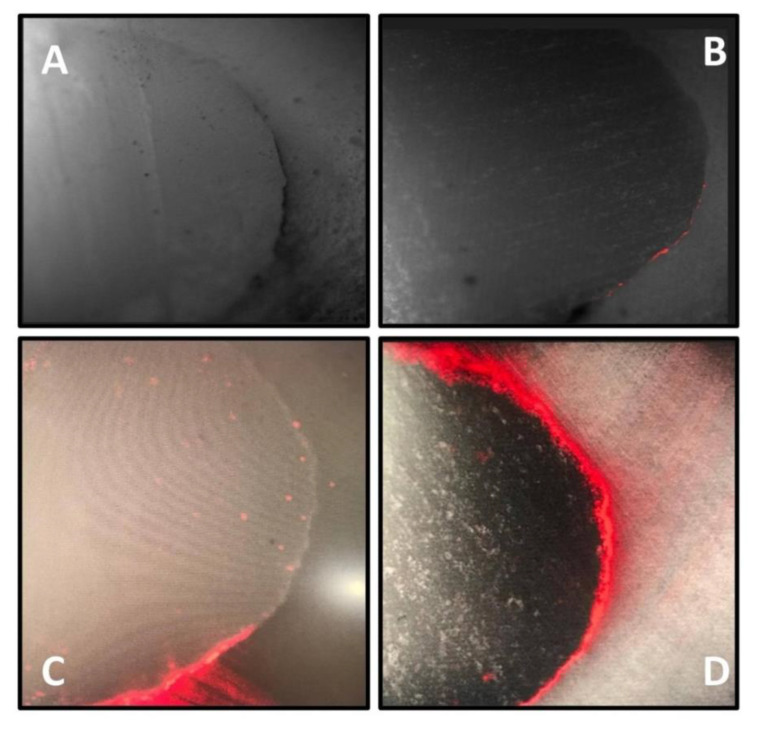
(**A**–**D**) Images of confocal laser microscopy showing scoring criteria with (**A**) Score 0; (**B**) Score 1; (**C**) Score 2; (**D**) Score 3.

The confocal microscopic evaluation of the samples in this study was carried out using the scoring system depicted in Figure 5. Microleakage was found in all of the materials tested. However, regarding the statistical analysis, the lowest amount of microleakage was seen in Adper Single Bond (ER-2 steps), which is a fifth generation bonding agent with a mean value (46.65), followed by G-Bond (SE-1 step), which is a seventh generation bonding agent with a mean value (51.69), and self-adhesive flowable composite resin (Constic), with a mean value (65.15). The difference in the microleakage of restorations carried out using the materials Single Bond and Constic was statistically significant.

The possible reasons for this result could be due to the fact that Single Bond is a fifth generation (ER-2 steps) bonding agent that comprises priming action and adhesive components of the conventional three-step adhesive system. It is composed of ethanol and water as a solvent. The presence of alcohol as a solvent has shown to increase the penetration into the dentinal tubules, thus aiding in adhesion. Moisture present in the dentinal tubules tends to draw the alcohol content into the dentinal tubules, which also takes resin along with it. Moisture and alcohol vaporize from the substrate and leave behind the resin content, thus providing a better bond strength [35].

The G-Bond showed a greater microleakage when compared with Single Bond, but this was not statistically significant. The possible reasons for this result could be due to G-Bond being the seventh generation (SE-1 step), and so working on a different mechanism of modifying the smear layer and not removing it; thus, the interface formed is different from the previously introduced adhesive systems. The decalcification of the dentin surface is less and there is almost no exposure of the collagen fibers. Hence, a very thin layer of the interface is formed, explaining the results obtained in this study. A nano level of insoluble calcium is formed due to the interaction between the functional monomers and the hydroxyapatite crystals. Thus, a strong bonding is expected from this bonding agent. This nano-level interaction is also termed the nano interaction zone or a “nano-level” interacted layer. These could be the reasons for better adaptation and microleakage values close to the gold standard etch and rinse system [33].

The current study results are in harmony with research carried out by Alfonso Sánchez-Ayala et al. to evaluate the microleakage in class V resin restorations bonded with six one-step self-etch systems and one etch and rinse adhesive. Seventy class V resin-based composite restorations were prepared on the buccal and lingual surfaces of 35 premolars by using: Clearfil S3 Bond, G-Bond, iBond, One Coat 7.0, OptiBond All-In-One or Xeno IV. The Adper Single Bond etch-and-rinse two-step adhesive was employed as a control. Specimens were thermocycled. None of the adhesives avoided microleakage at the dentin margins, and they displayed similar performances (*p* = 0.76). Their study showed that less microleakage was seen in Adper Single Bond compared to the G-Bond. They justified that Adper Single Bond 2 is able to wet and impregnate the etched enamel in an efficient manner comparable to those of three-step systems. However, its efficiency on dentin is lower because the adhesive incompletely diffuses under wet-bonding conditions, and a porous collagen network remains. Phase-separation also occurs in the interphase region between the hydrophilic primers and hydrophobic resins, resulting in water absorption. This effect may be justified by the incorporation of a high-molecular-weight polyalkenoic acid and the presence of HEMA. Nevertheless, the incorporation of alcohol as a cosolvent may help to explain its performance [11].

MitraTabari et al. conducted a study to evaluate the microleakage of composite resin restoration using a fifth generation bonding agent and two seventh generation bonding agents. This study was performed on 45 intact human-extracted primary teeth. Following class V cavity preparation, the samples were randomly divided into three groups, including 15 teeth based on the type of bonding agent: Single Bond 2, Clearfil S3 Bond, or G-Bond. There was no significant difference between incisal and gingival microleakage considering the different types of bonding [35].

In this study, Constic showed a greater amount of microleakage when compared with Single Bond and G-Bond. The possible reasons could be due to the incorporation of different monomers and compositions. A hard tissue interaction of self-adhesive flowable composites is found to be unlike the conventional composites [36]. The flowable composite tends to show a high water absorption. When the hydrophilic monomers are correlated to the conventional composites, they display a high tendency of water absorption. This, in turn, results in the breakdown of the polymer chains as the matrix swells due to water absorption because of the hydrophilic nature of the monomers in self-adhesive flowable composites. Such interactions might affect the bonding ability of the self-adhesives, which, in turn, cause a weak adhesion and failure [37].

Anshula Deshpande carried out an in vivo study to evaluate the retention rate, marginal integrity and marginal discoloration of two different sealants in which Constic and Conseal was used as a pit and fissure sealant. Constic showed a lower sealing ability when compared to Conseal. The Conseal-F sealant was better than the flowable composite as a sealant with respect to the marginal integrity and anatomical form. Both the materials showed similar results with respect to marginal discoloration [26].

However, a study carried out by Amey Panse compared the efficacy of the new material to the conventional sealant. Seventy-six noncarious primary molars were randomly assigned into two groups, Fissurit F (Group A) and Constic (Group B). Each group was further subdivided into four groups: G1—microleakage (*n* = 18), G2—fracture strength (*n* = 18), G3—tensile strength (*n* = 20), G4—shear strength (*n* = 20). The microleakage and fracture strength of Constic were found to be better, but the bond strength of Fissurit F (tensile strength—14.30 ± 4.49; shear bond strength—6.12 ± 2.84) was greater than that of Constic (tensile strength—6.33 ± 1.47; shear bond strength—2.06 ± 0.635). Their results showed Constic to be more fracture-resistant and presented less microleakage [38].

Hence, the null hypothesis for this study was rejected, as there was a difference in microleakage amongst the study groups, i.e., etch and rinse adhesive system ER-2steps (Adper Single Bond 2), self-etch adhesive system SE-1 step (G-Bond), and self-adhesive flowable composite resin (Constic) in Class V cavities.

The biocompatibility of these new products may be a matter of concern, but the dentin bonding agents and composite resins mentioned in this study are commercially available materials. Research by Pagano S et al. suggested that adhesives showed an effect on the functionality of fibroblasts, with a cytotoxic effect on time, that was concentration-dependent. The in-depth analysis of the effects of universal adhesives and possible functional effects represents important information for the clinician towards choosing the most suitable adhesive system [38].

Continued technological development in dental materials has led to the introduction of ‘bioactive materials’ that can activate dental tissue repair mechanisms, as well as elicit a positive response from the dental tissue. With characteristics similar to composites and glass ionomer cements, these can be especially useful in restorative dentistry. This could be future scope for scientific research in the ever-increasing clinical use of bioactive composites in the restoration of cervical lesions [39].

The limitation of this in vitro study is that the simulation of oral environment was not possible. There is a need for further in vivo research with regards to microleakage that compares the self-adhesive composite resin. Recently, different adhesive systems have been introduced in dentistry for better marginal adaptations and less microleakage. Their sealing ability should be assessed both in vitro and in vivo using different techniques.

## 5. Conclusions

None of the adhesive systems tested in the above study were free from microleakage. In the present study, the total etch adhesive ER-2 steps, i.e., fifth generation bonding agent (Adper Single Bond 2), showed the least microleakage values, followed by SE-1 step i.e., seventh generation bonding agent (G-Bond) and self-adhesive flowable composite (Constic). Constic showed the highest amount of microleakage.

The difference in microleakage between the self-adhesive flowable composite (Constic), etch and rinse (ER-2 steps) fifth generation (Adper Single Bond), and self-etch (SE-1 step) seventh generation bonding agent (G-Bond) was statistically significant. When etch and rinse (Adper Single Bond 2) and self-etch (G-Bond) were compared, more microleakage was seen in the G-Bond compared to Adper Single Bond 2, the difference not being statistically significant.

## Data Availability

Not applicable.
